# Natural Solid-State Hydrogel Electrolytes Based on 3D Pure Cotton/Graphene for Supercapacitor Application

**DOI:** 10.3390/mi14071379

**Published:** 2023-07-05

**Authors:** Nujud Badawi Mohammed, Khalid Mujasam Batoo, Sajjad Hussain, Ramesh Subramaniam, Ramesh Kasi, Mrutunjaya Bhuyan, Ahamad Imran, Muthumareeswaran Muthuramamoorthy

**Affiliations:** 1 Centre for Ionics University of Malaya, Department of Physics, Faculty of Science, Universiti Malaya, Kuala Lumpur 50603, Malaysia; lyde467@hotmail.com (N.B.M.); rameshkasi@um.edu.my (R.K.);; 2 Department of Physics, Faculty of Science, University of Hafr Al-Batin College of Science, Hafer Al-Batin 39921, Saudi Arabia; 3 King Abdullah Institute for Nanotechnology, King Saud University, P.O. Box 2455, Riyadh 11451, Saudi Arabia; aimran@ksu.edu.sa (A.I.); pharmapearll@gmail.com (M.M.); 4 Graphene Research Institute, Sejong University, Seoul 05006, Republic of Korea; shussainawan@gmail.com; 5 Institute of Nano and Advanced Materials Engineering, Sejong University, Seoul 05006, Republic of Korea; 6 Center of Theoretical and Computational Physics, Department of Physics, Universiti Malaya, Kuala Lumpur 50603, Malaysia

**Keywords:** cotton, conductivity, supercapacitor, graphene, energy

## Abstract

A conductive cotton hydrogel with graphene and ions can come into contact with electrodes in solid electrolytes at the molecular level, leading to a more efficient electrochemical process in supercapacitors. The inherently soft nature of cotton mixed with hydrogel provides superior flexibility of the electrolyte, which benefits the devices in gaining high flexibility. Herein, we report on the current progress in solid-state hydrogel electrolytes based on 3D pure cotton/graphene and present an overview of the future direction of research. The ionic conductivity of a complex hydrogel significantly increased by up to 13.9 × 10^−3^ S/cm at 25 °C, due to the presence of graphene, which increases ionic conductivity by providing a smooth pathway for the transport of charge carriers and the polymer. Furthermore, the highest specific capacitance of 327 F/g at 3 mV/s was achieved with cyclic voltammetry measurement and a galvanostatic charge–discharge measurement showed a peak value of 385.4 F/g at 100 mA/g current density. Furthermore, an electrochemical analysis demonstrated that a composite cotton/graphene-based hydrogel electrolyte is electrically stable and could be used for the design of next-generation supercapacitors.

## 1. Introduction 

Nowadays, flexible energy storage devices, such as supercapacitors, have received wide attention as they offer flexible and wearable electronics. Solid-state supercapacitors are new types of supercapacitors that can operate under successive expansion, bending, and torsion conditions. However, many challenges are encountered to obtain a flexible supercapacitor in the solid state with high electrochemical performance and super flexibility. The performance of flexible supercapacitors (SCs) depends on the properties of the electrolyte materials, electrode materials, and device configuration [1]. Flexible solid-state super capacitors (FSSSC) are characterized by fast charge and discharge rates, high energy density, ease of manufacture, and low cost, and have promising applications as power sources for multifunctional portable and wearable electronic devices [2]. The mechanical strength of the active electrodes and the electrolyte is what determines the configuration and flexibility of the devices; thus, supercapacitors are expected to have excellent mechanical flexibility and to achieve the absorption of significant levels of stress in rigorous real-world applications [3]. Carbon materials with variable and varied microstructures present great opportunities for the manufacture of high-performance flexible active-electrolyte FSSSCs. For example, graphene is a two-dimensional carbon material that has extraordinary physical properties, such as a large specific surface area, strong mechanical strength, high optical transparency, ultra-fast electronic mobility, and excellent thermal conductivity; therefore, it is an ideal active material for supercapacitors [4]. However, owing to the p–p stacking interaction and van der Waals force, graphene is easy to agglomerate [5].

Aqueous electrolytes have attracted much attention due to several reasons. Aqueous electrolytes do not need the introduction of water and oxygen, such as organic electrolytes do, which greatly reduces the cost. Moreover, they have low viscosity, low resistance, and high safety. The smaller the resistance of ion transport, the lower the thermochemical temperature, which can accelerate charge storage and the process, thus improving device integrity. Importantly, compared to organic electrolytes, aqueous electrolytes have much smaller ion sizes and higher ionic conductivity, which gives them a greater capacity, superior charge and discharge, and excellent power performance. On the other hand, strong acidic electrolytes, such as H_2_SO_4_ and KCl with lower H^+^, generally have the highest ionic conductivity (0.8 S/cm) [6].

Cellulose, the first and most abundant renewable biopolymer in nature, can be obtained mainly from plants, such as cotton, wood, bamboo, and grass. Cotton is an inexpensive natural product, mainly based on cellulose, and is widely used in apparel and textiles in our daily life. Although cotton is an insulator, it is directly converted into carbonated cotton when treated with a conductive material, which makes it highly conductive while maintaining its flexibility, has excellent mechanical properties and structural compatibility, and with its improvement, the energy storage capacity of cotton-based supercapacitors (SC) becomes an important factor for practical applications [7].

Due to their unique qualities, including high mechanical strength, exceptional structural flexibility, high thermal and electrical conductivities, and high surface area, carbon-based e-textiles are an excellent substitute [8]. Due to their exceptional combinations of electrical, mechanical, and chemical properties compared to those of commercial e-textile technology, graphene-based textiles are considered particularly promising for use in materials and devices among the investigated carbon e-textiles used for smart fabrics and wearable devices [9]. They can be used as soft actuators, flexible supercapacitor electrodes, flexible wearable physical and chemical sensors, and multifunctional e-textiles like conducting cotton [10]. 

**Figure 1:**
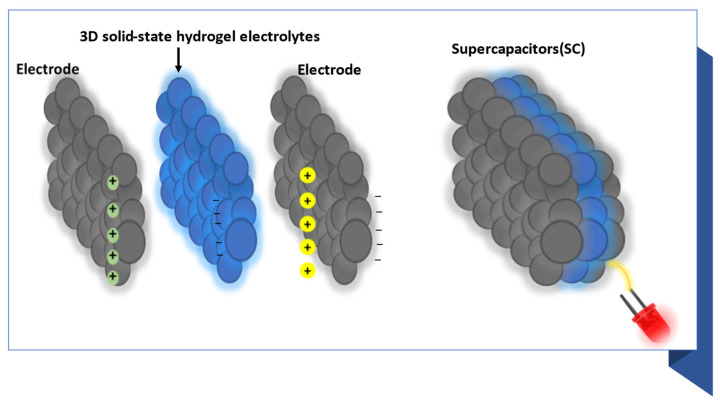
Supercapacitors based on 3D solid-state hydrogel electrolytes in bule and electrodes (pure cotton/graphene)in black color.

Here, the method of the controllable fabrication of cotton-based and graphene-based tandem solid-state hydrogel electrolytes, and the electrochemical/mechanical performance of graphene-based flexible FSSSCs (those based on cotton films and fibers), are associated with strategies commonly used to improve performance. The physical properties of the fabricator membranes and electrochemical performances of the SCs have been marked. In the final part, this report concludes with some challenges and prospects for the use of flexible graphene/cotton-based FSSSCs as power systems for portable and wearable electronics through LED lighting. Figure 1 shows a sandwich of two electrodes made of cotton/graphene, connected by a flexible hydrogel of cotton treated with graphene. After electrical conduction, the LED was lit.

## 2. Materials and Preparation

### 2.1. Raw Materials

**Figure 2:**
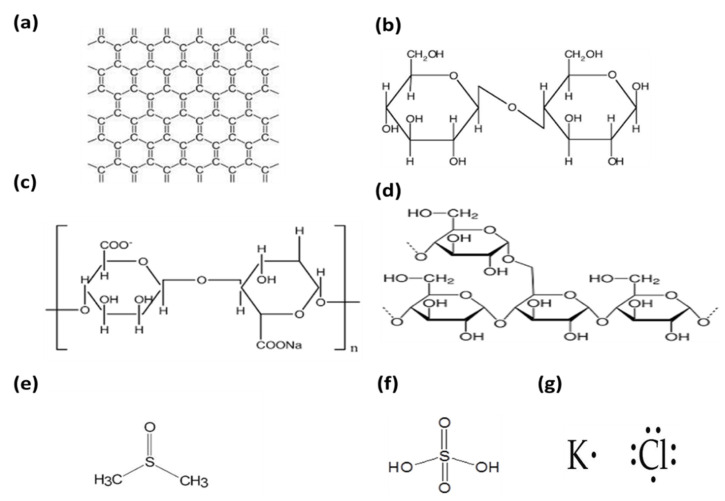
The chemical formula for (**a**) graphene, (**b**) cotton, (**c**) sodium alginate, (**d**) starch, (**e**) dimethyl sulfoxide DMSO, (**f**) sulfuric acid (H_2_SO_4_), and (**g**) potassium chloride (KCl).

Cotton (100% pure natural) and graphene powder (G) were purchased with an initial flake size of 100 mesh from Sigma Aldrich (Gillingham, UK) (100% pure). Raw materials, such as sodium alginate powder (M_n_ = 357,475, M_n_/M_w_ = 1.392, and M/G = 0.32), starch, dimethyl sulfoxide (DMSO), which lowers the surface tension and is good at dispersing graphene, (CH_3_)_2_SO (assay 99.9%), sulfuric acid (H_2_SO_4_), and potassium chloride solution (KCl) were purchased from Sigma Aldrich, Gillingham, UK. Deionized water (DI) was used as a solvent and the graphene-conducting substrate was used as an electrode substrate material. The chemical formulas of the materials used are shown in Figure 2.

### 2.2. Material Preparation

**Figure 3:**
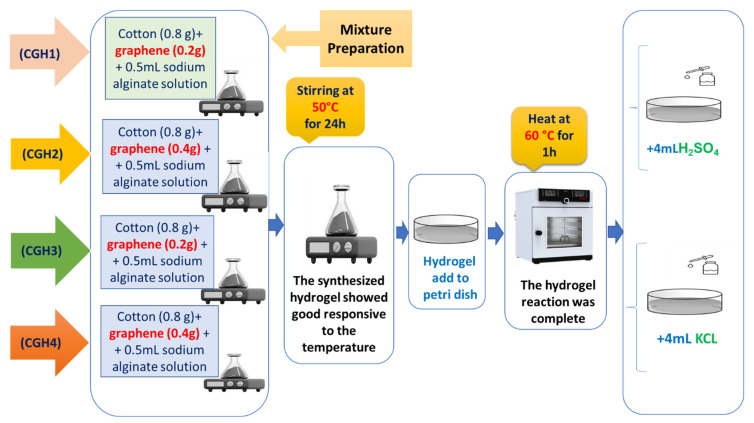
Solid-state hydrogel electrolytes preparation.

**Figure 4:**
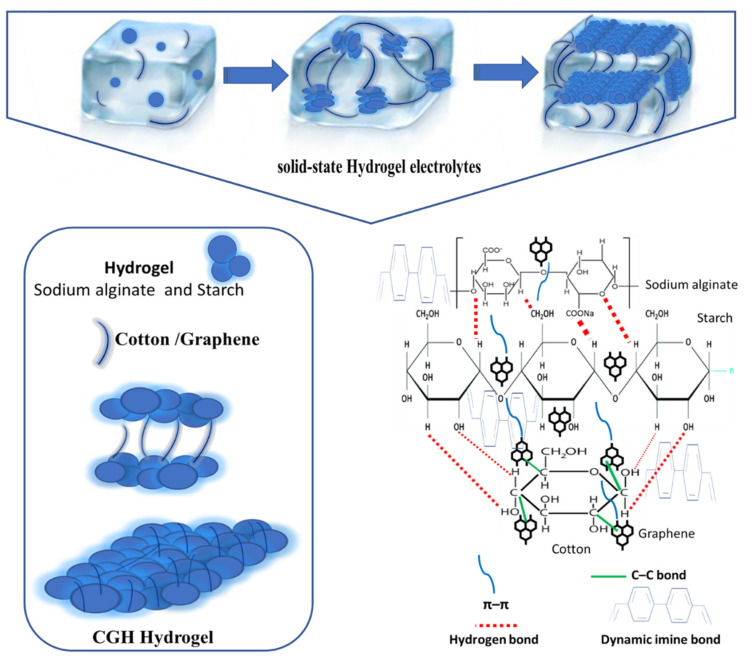
Schematic diagram of the preparation and mechanism of a highly flexible and mechanically stable hydrogel.

Figure 3 presents the stepwise preparation of the samples of the studied material. To prepare the hydrogel, sodium alginate (2 g) and starch were added to 100 mL water solution in a beaker using the hydrothermal method. Amounts of 0.8 g of cotton and 0.5 mL of DMSO were added to the prepared hydrogel and were stirred on a magnetic stirrer at 50 °C for 24 h. The solution was obtained after ultrasonic treatment for 5 min under ambient conditions. Graphene was then added to the above solution at 50 °C. The membranes were made with a simple solution-casting method. The samples were then heated in an air oven at 60 °C for an h. Once the oven was cooled down to room temperature, the corresponding cotton hydrogel was prepared by immersing the membranes in 4 mL KCl and H_2_SO_4_ solution. The samples were coded as CGH1, CGH2, CGH3, and CGH4, respectively, based on the cotton-to-graphene ratio, and the details are provided in Table 1. The polymerization reaction was completed after 1 h and the samples were freeze-dried before further characterization. Figure 4 presents the schematic diagram of CGH. Hydrogen bonds are formed by starch and cotton to improve the mechanical strength. Graphene plays a remarkable role as a supporting material for the fabrication of hydrogels [11,12]. Graphene can effectively cross-link the polymer chains of hydrogel through imine bonds, so that only the imine bonds are cleaved when the hydrogel solution and cotton are attached to the graphene structure, where the composite hydrogel is covalently bonded to graphene. Graphene platforms form in hydrogel by π–π stacking, because of the delocalized π electrons on the surface of graphene and, moreover, because of the C–C bond between graphene and cotton. Adding natural fibers, such as cotton, is an easy option that can generate hydrogels that are more resistant to impact and handling; the interaction with hydrogen bonds tends to be strong, which increases the compatibility between the cotton and the hydrogel.

### 2.3. Fabrication Supercapacitors

At ambient conditions, pure cotton/graphene solid-state hydrogel electrolytes were soaked in H_2_SO_4_ (CGH1 and CGH2) and KCl (CGH3 and CGH4) for 1 h. The hydrogel was directly cut into a size of 1.5 cm × 1.5 cm and used as the electrolyte.

Then, the SC was assembled in a coin cell with two identical electrodes based on graphene-treated cotton. The fabricated supercapacitor will perform as an electric double-layer capacitor (EDLC), since the 2 electrodes are carbon-based and do not involve any faradaic reactions. Similarly, the control sample was collected using a cotton-based electrolyte separator mixed with hydrogel. All operations were performed at room temperature, where two cotton electrodes were treated with graphene and pressed together face-to-face to assemble flexible solid-state SCs [12].

### 2.4. Characterization

The structural characterization of the hydrogel electrolytes was performed through the X-ray diffraction technique using an Empyrean diffractometer with CuKα (λ = 1.5406 Å) radiation, having a current of 30 mA and a voltage of 40 kV. The surface morphology was studied through field-emission scanning electron microscopy (FESEM, Japan Electron Optics Laboratory (JEOL) (Model: JSM-7600F), Tokyo, Japan), operating at an accelerating voltage of 5 kV. The samples were coated with gold before FESEM analysis.

An electrochemical impedance spectroscopy (EIS) was performed using a Hioki 3532-50 LCR HiTESTER impedance spectroscopy instrument in the frequency range from 50 Hz to 1 MHz. The ionic conductivity was calculated according to Equation (1) [13].

(1) $$\sigma =\frac{d}{R_{b}S}$$

where *d* is the thickness (cm) of the membrane, *S* is the surface or contact area (cm^2^), and *R_b_ is* the bulk resistance (Ω). 

The activation energy for ion transportation was calculated according to Equation (2) [14].

(2) $$\sigma =\sigma_{0}e_{RT}^{E_{a}}$$

where (*σ*) is the ion conductivity, (*σ*_0_) is the pre-exponential factor, (RT) where *R* is the ideal gas constant, and *T* is the temperature. ($E_{a}$) is the movement of a charge in response to an electric field. 

The ionic conductivity also depends on several factors: the nature of the electrolyte added, the nature of the solvent used and its viscosity, the concentration of the electrolyte, the size of the ions produced, their dissolution, and the temperature. The ionic conductivity also depends on several factors: the nature of the electrolyte added, the nature of the solvent and its viscosity, the concentration of the electrolyte, the size of the ions produced and their dissolution, and the temperature [14,15].

The electrochemical performances of the hydrogel electrolyte were studied through an electrochemical workstation using a Gamry Interface 1000 at different scan rates. Cyclic voltammetry (CV) and galvanic charge/discharge (GCD) curves were recorded for the voltage in the range of 3–100 mV/s and current densities from 100 mA/g to 500 mA/g, respectively. Specific capacitance values (Cs) were calculated from the CV and GCD curves using Equations (3) and (4) [16].

(3) $$C=\frac{\int ivdv}{2\mu m∆V}$$

(4) $$C=\frac{It}{m∆V}$$

Here, *v* represents the potential (*V*), *μ* represents the scan rate (V/s), *m* is the mass of active materials (g), and Δ*V* is the potential window of discharge (0.5 V here). The quantity, *I*, represents the constant discharge current (A), while *t* represents the discharge time (s).

The specific capacitance, power density, and energy density were calculated based on the galvanic charging–discharging curves using the equations [16,17].

(5) $$E_{g}=\frac{1}{2c∆v^{2}}$$

(6) $$P_{g}=\frac{E_{g}}{∆t}$$

where *C* represents the specific capacitance (Fg^−1^), *E_g_* is the energy density (Whkg^−1^), *P_g_* is the power density (Wkg^−1^), ∆𝑉 is the potential window (here 1.6 V), I is the discharge current (A), ∆𝑡 is the discharge time (s), and m is the sum of the masses of the positive electrode and negative electrode (g). The volumetric energy density can be estimated by using Equations (7) and (8) [18].

(7) $$E_{v}=\frac{E_{g}m}{2\;\mathrm{cm}\;2\;\mathrm{cm}\;0.05\;\mathrm{cm}}$$

(8) $$P_{v}=\frac{P_{g}m}{2\;\mathrm{cm}\;2\;\mathrm{cm}\;0.05\;\mathrm{cm}}$$

The theoretical capacitance of the asymmetric full cell was calculated according to Equation (9) [19].

(9) $$\frac{1}{C_{T}}=\frac{1}{C_{P}}+\frac{1}{C_{N}}$$

where *C_T_* is the total capacitance of the cell, *C_P_* is the capacitance of the positive electrode, and *C_N_* is the capacitance of the negative electrode.

Cell capacitance is best determined from the galvanostatic or constant current (CC) discharge curves using Equation (10) [20].

(10) $$C=\frac{1}{4}\frac{IdV}{dt}\;$$

Here, *dV/dt* is calculated from the slope of the CC discharge curve. Galvanostatic discharge is the accepted measurement method for determining the capacitance of packaged ultracapacitors in the ultracapacitor industry and correlates more closely to how a load is typically applied to an ultracapacitor in the majority of applications [20,21].

A Fourier transformation infrared spectroscopy (FTIR) study was carried out using a Thermo Nicolet Avatar 380 FTIR spectrometer equipped with an attenuated total reflection (ATR) attachment, with a germanium crystal in a frequency range from 4000 to 500 cm^−1^ and with a spectral resolution of 1 cm^−1^. This measurement was performed to reveal the complexity of the solid-state hydrogel electrolytes host, the presence of specific functional groups and free ions, and to suggest conduction mechanisms [22]. 

Tensile testing was performed using an Instron 3360 electronic universal testing device (Instron Corporation, Norfolk County, MA, USA) on the CGH1, CGH2, CGH3, and CGH4 hydrogels, respectively. The prepared specimens were cut into rectangular shapes according to the JISK6251-7 standard size (length 2.5 cm, width 0.6 cm, and gauge length 0.6 mm).

## 3. Results and Discussion

### 3.1. X-ray Diffraction (XRD) Analysis 

**Figure 5:**
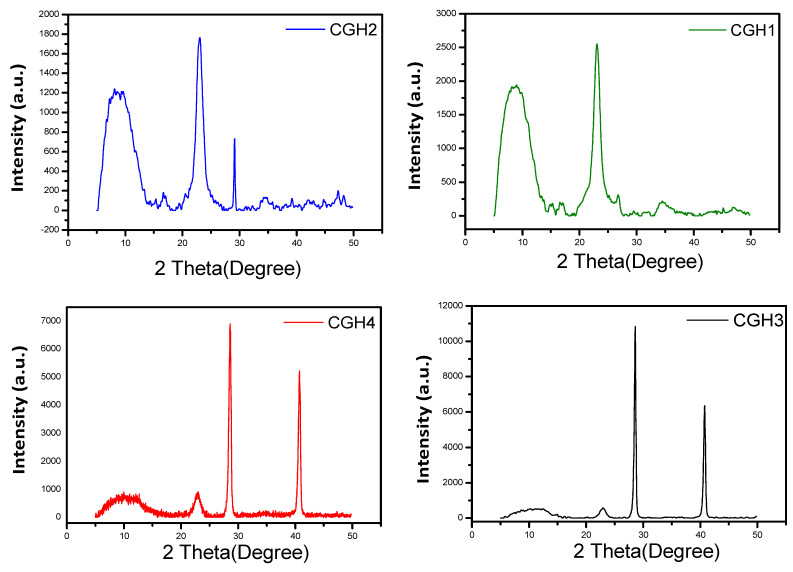
XRD patterns for the samples CGH1, CGH2, CGH3, and CGH4.

In Figure 5, all the CGH hydrogel electrolyte materials are depicted with their XRD patterns. According to the XRD patterns of the CGH1 and CGH2 hydrogel electrolytes, these electrode materials are highly crystalline in nature [23]. The diffraction peaks of the CGH2 hydrogel electrolyte observed at the 2θ = 8.6°, 10.6°, 14.7°, 18.2°, 20.0°, 23.5°, 25.2°, 27.5°, 30.5°, 33.2°, 35.4°, 36.6°, 37.5°, 39.2°, 40.4°, 41.1°, 43.6°, and 45.1°, correspond to the crystal planes (1 1 0), (0 2 0), (2 0 0), (−1 0 1), (0, 1, 1), (1 3 0), (1 0 1), (0 3 1), (−3 0 1), (−1 4 1), (3 3 0), (1 4 1), (3 0 1), (−1 1 2), (−3 4 1), (−2 5 1), (3 5 0), and (1 3 2). In contrast, the diffraction peaks observed at 6.5°, 10.5°, 15.5°, 23.5°, 28.2°, 35.5°, and 41.8° are indexed according to the crystal planes (1 110), (0 20), (2 20), (1 001), (0 311), (1 41), and (−251). 

Both the XRD patterns of CGH1 and CGH2 had almost similar peak positions. This is attributed to the treatment of the hydrogel electrolytes with H_2_SO_4_, as they had an approximately similar scattering factor [23,24]. Consequently, the crystal planes of the CGH3 and CGH4 hydrogels electrolytes are also the same. The hydrogel electrolytes (CGH1, CGH2, CGH3, and CGH4) also show sharp peaks in their XRD patterns, indicating their high crystallinity [24].In addition, the CGH1 hydrogel electrolytes show that the changes in the CGH2 and CGH4 hydrogel electrolytes, with high concentrations of graphene, affect the XRD peak positions. Hence, when comparing the XRD patterns of the CGH hydrogels electrolyte materials (CGH1, CGH2, CGH3, and CGH4), the higher the graphene content, the higher the intensity peak. The strong diffraction peaks observed at 2θ = 8.5°, 9.6°, 22.9°, and 34.5° are characteristic peaks of cotton and starch that correspond to the planes indexed for Miller indices (110), (110), (102), and (200), respectively. Graphene exhibits a strong X-ray peak at 2θ = 23.5° [25].

### 3.2. FTIR Analysis

**Figure 6:**
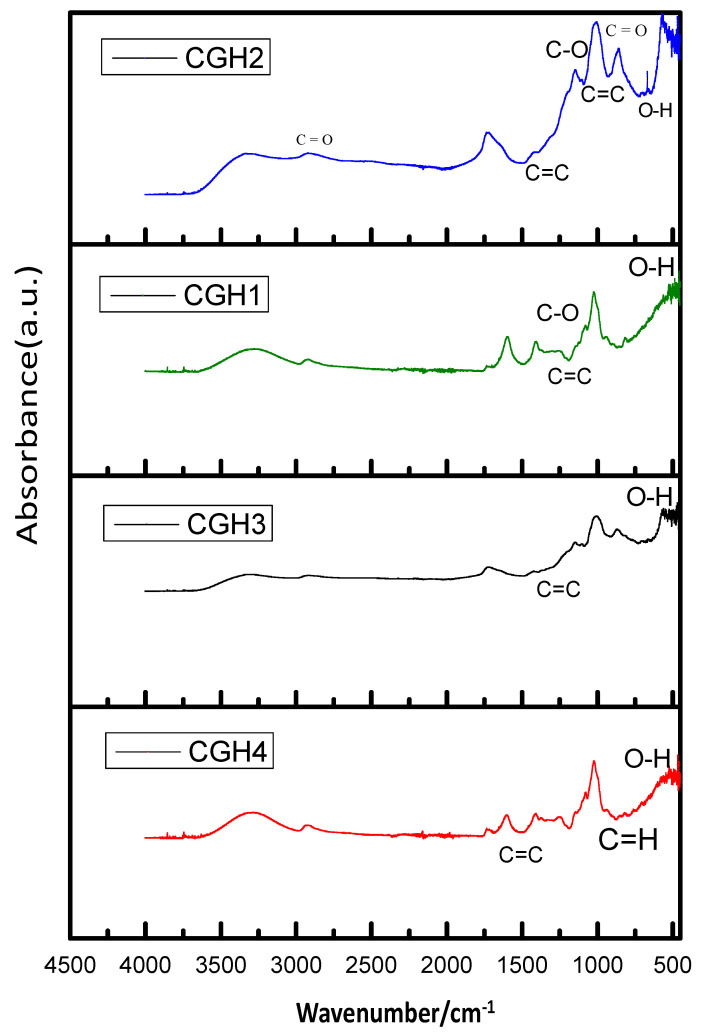
FTIR spectroscopy for the samples CGH1, CGH2, CGH3, and CGH4.

Figure 6 shows the FTIR spectra of hydrogels based on 3D pure cotton/graphene. The FTIR spectra of the CGH1 and CGH3 hydrogels show a weak absorption band at 1700 cm^−1^ due to the expansion vibration of C=O. The band observed at 1227 cm^−1^ is associated with the C–O groups and the band at 1045 cm^−1^. However, the broad band observed at 1000 cm^−1^ is due to the O–H expansion mode [25]. This is because there are still few oxygen-containing groups on the surfaces of CGH2 and CGH4 in comparison to the samples treated with high concentrations of graphene. The cotton sample shows a peak at 3500.55 cm^−1^, corresponding to the O–H stretching vibration mode, and the bands observed at 1620.77 cm^−1^, 1402.33 cm^−1^, and 1360.11 cm^−1^ correspond to the C–H stretching vibration, O–H bending vibration, and C–O stretching vibration modes. The peaks observed at 2870 cm^−1^ and 570 cm^−1^ in the sample CGH2 appear to be due to treatment with H_2_SO_4_ [25,26]. The hydrogel presents new peaks which are attributed to graphene, C=C, which causes an extended deformation at 1500 cm^–1^. The oxygen stretching vibrations were found near 1450 cm^−1^ in all the hydrogels’ spectra. The group C=C results in an extended deformation at 1355 cm^−1^ and C–H aromatic bends at 500 cm^–1^ [26,27].

The FTIR analysis is very well in agreement with the XRD results and hence provides indications of the positions of the absorption bands and the spinel phase frequency, respectively.

### 3.3. Morphological Studies

**Figure 7:**
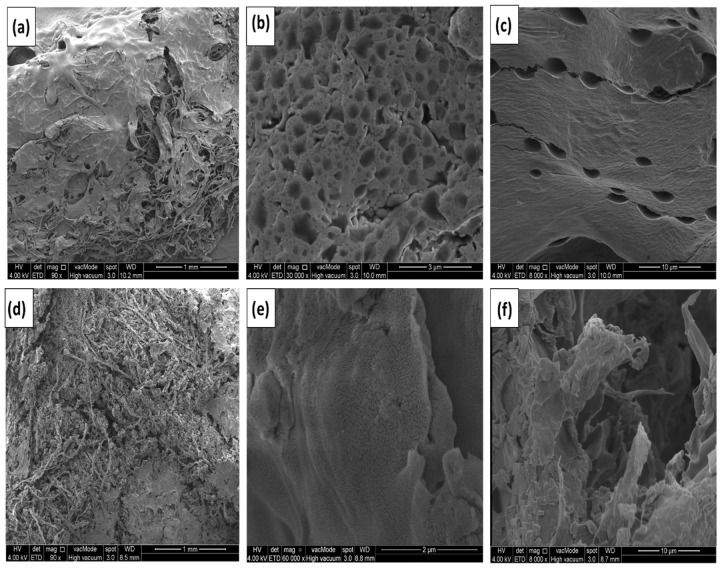
SEM images (**a**–**c**) of CGH1 and (**d**–**f**) of CGH2.

**Figure 8:**
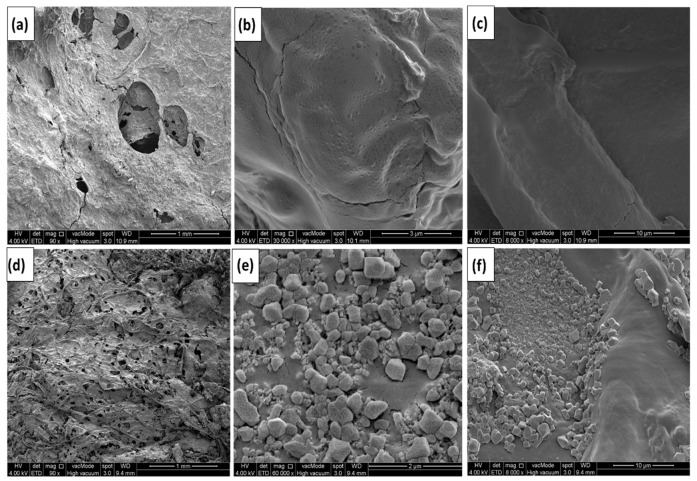
SEM images (**a**–**c**) of CGH3 and (**d**–**f**) of CGH4.

An FESEM study was carried out to inspect the surface morphology of the hydrogel electrolytes, as shown in Figure 7 and Figure 8. Graphene formation/composition ratios are seen to be relatively higher for samples CGH2 and CGH4 when compared to the morphology of CGH1. There is, however, a correlation between the amount of graphene in the hydrogel and the amount of pore distribution in the films of the samples CGH2 and CGH4, as well as an increase in pore size when the graphene content is increased. As indicated by the higher absorption, due to the presence of cotton in the hydrogel formation when graphene is present [28,29], this may be due to phase separation between the graphene and compound hydrogel. Phase separation occurs when chain length and surface area increase, indicating the presence of graphene in the hydrogel formation. This study examines the microstructures of the as-prepared samples CGH1, CGH2, CGH3, and CGH4 in different combinations, as shown in Figure 7b,c and Figure 8e,f. In such a way, the fiber bundle structure is destroyed and the graphene rods are attached to it. This creates a three-dimensional network structure that is characterized by a high number of pores and an almost uniformity in their sizes and surfaces [20,29]. The 3D porous structure of the electrode enables the rapid transfer of ions from the electrolyte to the entire surface of the electrode, where there can be a rapid formation of an electrical double layer [30]. The microstructure of the sample CGH2 can be seen in Figure 7d, which shows long fibers intertwined with each other, forming a network structure with a large number of cavities with the hydrogel, in which a large number of cavities contribute to high electrolyte liquid absorption [31]. In addition, it is observed that the membrane is a film consisting of a dense hydrogel evenly distributed over the surface of the graphene-treated fibers; in addition, the cotton fiber bundle reveals a cross-linked structure that forms a three-dimensional network of cotton [32]. It can be seen that crystallization of the sample occurs uniformly when immersed in H_2_SO_4_ (CGH1 and CGH2), while hydrogel clumps form on the surface when the sample is immersed in KCl (CGH3 and CGH4), as shown in Figure 8d,e. Furthermore, the FESEM image of sample CGH2 is shown in Figure 7d, which shows that the obtained active hydrogel layers show a high homogeneous particle dispersion as the cotton/graphene fabric coats it uniformly, due to the H_2_SO_4_ treatment [33,34].

### 3.4. Electrochemical Impedance Performance of Hydrogel Electrolytes

The ionic conductivity of the composite hydrogel electrolytes CGH1, CGH2, CGH3, and CGH4 was determined at room temperature. For all synthesized hydrogel electrolytes, graphene is the main component that makes ionically conductive hydrogels. Furthermore, the addition of DMSO enhances ionic conductivity, as it supports ionic diffusion through the cotton networks as the highly porous 3D structure increases [34,35]. Cotton’s higher water absorption potential leads to smoother ways of transporting ions across the polymer network [35]. The ionic conductivity is increased through the smoothing of the electrolytic ion flow by the water molecules around the charged groups [36].

**Figure 9:**
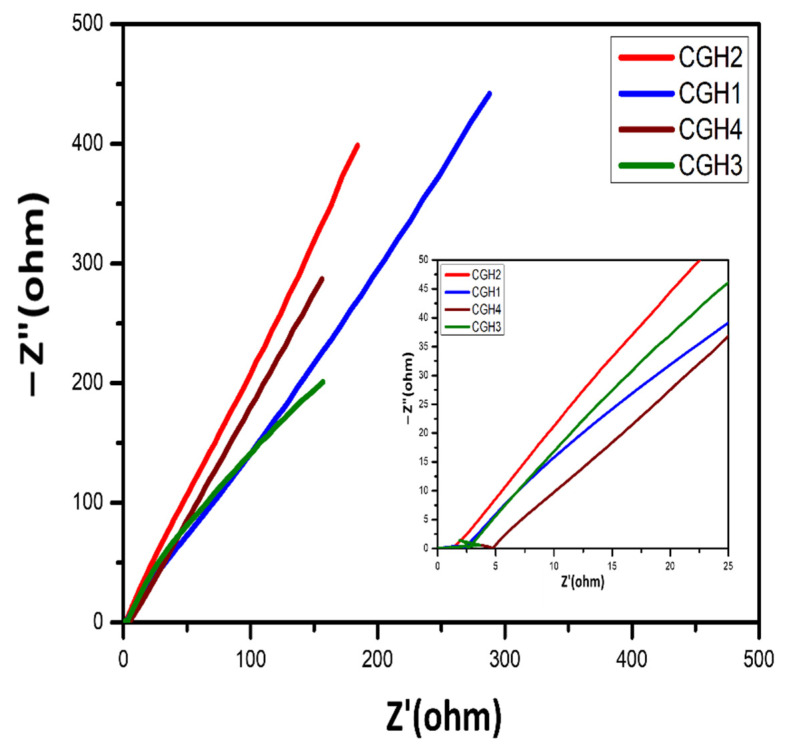
Nyquist plots for the samples CGH1, CGH2, CGH3, and CGH4 at room temperature.

Figure 9 shows the Nyquist plots for the hydrogel electrolytes which indicates the capacitive behavior, with less resistance at a higher frequency and a steeper slope at a lower frequency for the prepared samples. In the Nyquist plot, at 45° a straight line begins immediately from the ESR, which is a typical feature of a porous electrode, followed by a line that is parallel to the Z″-axis in the lower frequency region [36]. The straight line at a higher frequency indicates the pure capacitive nature of the hydrogels, due to the rapid transport of the charge carriers at room temperature. It is seen that the ion transfer is smooth for the charge carriers due to the available free space that is achieved through stretching. The ionic conductivity was determined for the cotton hydrogel samples CGH1, CGH2, CGH3, and CGH4, respectively, at 11.5 × 10^−3^, 13.9 × 10^−3^, 10.9 × 10^−3^, and 8.7 × 10^−3^ S/cm, depending on the ionic concentration [37,38,39]. It is seen that a higher value of impedance is noted for the sample CGH4, and the resistance of sample CGH1 increases when the chain length of the synthesized hydrogel increases. The sample CGH4 exhibits the lowest ionic conductivity of 8.7 × 10^−3^ S/cm due to its low porosity and semi-crystalline structure, which is due to the agglomeration of materials. The ionic conductivity of samples CGH3 and CGH4 decreases as the chain length increases due to their reduced porosity, whereas samples CGH2 and CGH1 have higher ionic conductivities of 13.9 × 10^−3^ S/cm and 11.5 × 10^−3^ S/cm, because of their porosity and electrolyte absorption.

### 3.5. Cyclic Voltammetry (CV) 

**Figure 10:**
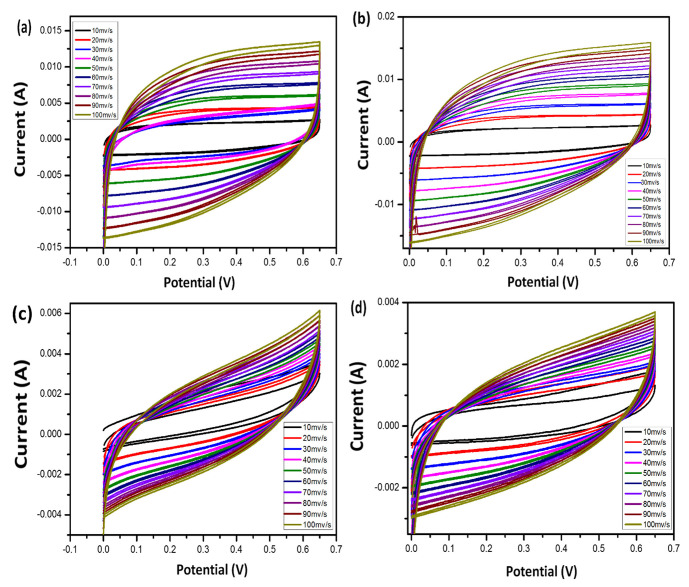
(**a**–**d**) CV curves at different current densities for CGH1, CGH2, CGH3, and CGGH4.

As shown in Figure 10, the CV measurements were conducted in the potential range of 0–1.6 V at a scanning rate of 10, 20, 50, and 100 mA/g, to determine the potential of the prepared cells. There is no apparent peak in the CV curves, indicating that the supercapacitor is charging and discharging at a constant rate [40]. Sample CGH2 shows a much higher CV region than the other samples for all scan rates, which could be explained by the fact that as the samples are soaked in H_2_SO_4_, the number of free water molecules decreases, transforming the electrolyte into a gel, which only allows a small number of free ions to enter the electrode micropores.

**Figure 11:**
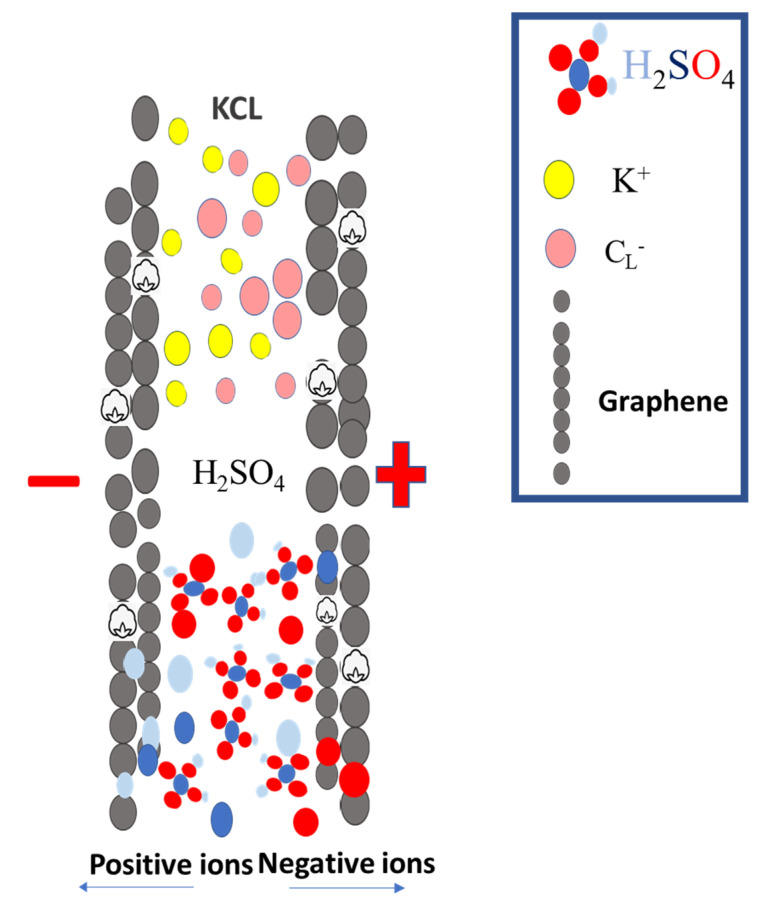
Schematic illustration of ion diffusion in different electrolytes.

**Figure 12:**
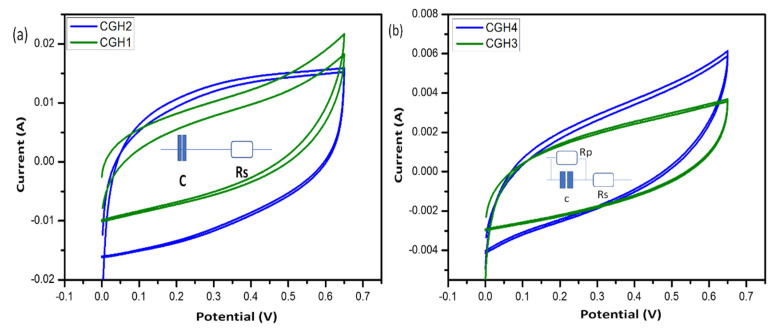
The shape of CV curves represents the capacitive response for the samples (**a**) CGH1, CGH2 and (**b**) CGH3, CGH4, with an inset showing the equivalent circuits representing the typical behavior of the samples.

The presence of graphene results in a significant increase in capacitance as a result of this. As the scanning rate of the cotton–graphene-based hydrogel is increased, the CV curves of the hydrogel begin to resemble a rectangular shape, even at 100 mA/g, when the CV curve is scanned at a higher rate. As shown in Table 2, there are no obvious oxidation peaks in the scanning range, suggesting a superior capacitive behavior [41]. Their specific capacitances are higher than those of the samples CGH1, CGH2, CGH3, and CGH4, with specific capacitances of 312.88, 390.75, 250.30, and 333.50 F/g, respectively. As a result, of these energy density values, 48.79, 50.99, 35.18, and 52.25 Wh/kg at a power density of 478.50, 49.99, 100.00, and 100.10 W/kg, respectively, are also high in terms of energy density. With increasing graphene ratios, the electrochemical stability window of the electrolyte improves with increasing capacitance, and no decomposition reaction peak is observed, even at 1.6 volts. The reason for this is due to the high porosity, electrolyte absorption, and hydrogen bonding with water molecules [42]. The composite hydrogel sample, which contains CGH1 as the effective electrode material, behaves in a manner that results in low electrical conductivity, as observed from the ideal cyclic behavior observed during CV measurements, with a pattern of charge and discharge cycles that resembles those in Figure 11. The hydrogel treated with KCl has a much smaller number of ionized ions compared to the H_2_SO_4_-treated hydrogel, since H^+^ ions can easily and rapidly diffuse between the layers of graphene and cotton as compared to the heavy K^+^ ions. It should be noted that only the oversized surface of the graphene electrode can be accessed during charging and discharging. Meanwhile, H_2_SO_4_ can ionize more free ions than KCl at the same molar concentrations. In a double-layer capacitor, the negatively charged (−) electrode attracts positively (+) charged ions and vice versa; the H_2_SO_4_ ions penetrate the electrode while the ions in KCl cannot penetrate the electrode, as shown in Figure 11. Thus, the supercapacitor using H_2_SO_4_ shows a specific capacitance higher than the basic capacitance. Figure 12 describes the circuit model of a periodic voltmeter. The central idea is to represent a non-ideal circuit consisting of a capacitor (C) connected in parallel with a resistor (Rp) and a series resistor (Rs), respectively, to illustrate the role of each circuit parameter (inset Figure 12) [42]. Furthermore, we observed a unique porous cotton-fiber structure within the hydrogel blend. A heat treatment with H_2_SO_4_ and KCL can be used to better control the internal structure and to adjust the properties of the formed structures of the supercapacitor, while the ESR can measure the transition between the electron spin energy levels. The rectangular-shaped plots turn out to be blunt CVs due to the presence of ESR (Figure 12a), which are suitable for supercapacitors but become weak and italic with both EPR and ESR, as shown in Figure 12b, which indicates that the capacitive area decreases. In models of electrolytic capacitors CGH3 and CGH4, the CVs appear slightly deviated from the ideal behavior due to ESR and EPR, in which ESR represents the finite resistance of the graphene-treated cotton electrode material, whereas EPR represents the ohmic conduction through the capacitor via an electrolyte gel [43].

**Figure 13:**
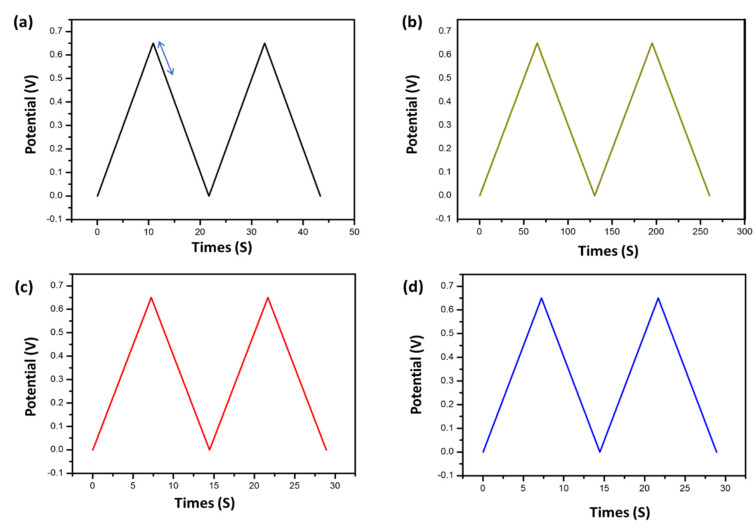
The panels (**a**–**d**) are for the galvanostatic charge–discharge (GCD) curves at 100 mA/g for the samples CGH1, CGH2, CGH3, and CGH4, respectively.

In addition, GCD was implemented at current densities of 100 mA for the samples CGH1, CGH2, CGH3, and CGH4, which show symmetrical triangles with different current densities, as seen in Figure 13a–d, indicating the energy storage mechanism is a typical capacitive type of EDLC. By applying a voltage, the ions are driven to the surface of the electrolytic double layer and the supercapacitor is charged. Inversely, they move away when the supercapacitor is discharged and the hydrogel is also responsible for preventing or shortening the connection of the AC electrodes. The curve is almost linear within the entire potential range, which shows the excellent performance of the four supercapacitors [44]. The symmetrical GCD curves indicate that the charge and discharge processes of the electrode materials are highly reversible [45]. Electrolytic double-layer capacitors (EDLCs) have a higher capacity than previous generations of dielectric capacitors, due to the composition of molecularly thin double-Helmholtz layers throughout the porous networks of cotton and graphene hydrogel electrodes, where hydrogels have precisely defined surface areas [46]. The electrical response generated by EDLCs is similar to that of dielectric capacitors. However, the surface functional groups on graphene can lead to parasitic electrochemical reactions, which lead to deviations from the ideal triangle for loading and unloading appearance [47]. We noted that the four devices behaved in an ideal manner similar to commercial capacitors, where redox dopants store electrical charge via electron transfer or Faraday interactions and the charging time is the same as the discharging time, since the current is the same for charging and discharging but in the opposite direction.

CGH2 is considered to be the best gel electrolyte for supercapacitors (large CV area, highest capacity reaction, and highest electrical conductivity). For the preparation of gel electrolytes for devices, sulfuric acid H_2_SO_4_ offers an efficient strategy [48].

### 3.6. Tensile Strain

**Figure 14:**
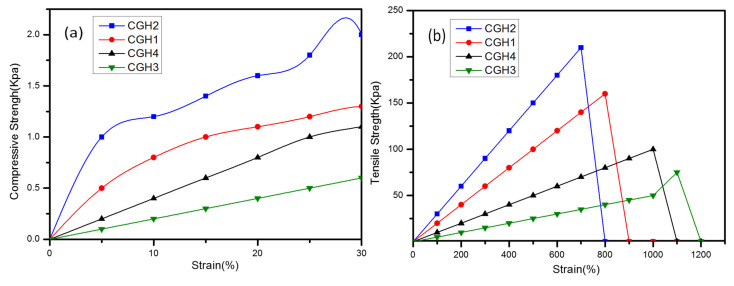
(**a**) Typical compressive strain–stress curves for CGH1, CGH2, CGH3, and CGH4, respectively. (**b**)Typical tensile strain–stress curves for CGH1, CGH2, CGH3, and CGH4.

To characterize the mechanical properties of the hydrogels CGH1, CGH2, CGH3, and CGH4, both compression and tensile tests were performed. Hydrogels exhibit brittle fractures due to their stress–strain behavior, which is linear and reaches the breaking point before decomposing. At compressive deformations of 34% and 36%, respectively, the compressive strength of the CGH1 and CGH3 hydrogels reached 0.0056 and 0.0059 MPa. Despite this, the CGH1 hydrogels maintained good ductility about two times higher than the CGH3 hydrogels, in terms of elongation at breakage [49]. When graphene was added to the CGH4 hydrogel, its compressive strength increased to 0.16 MPa at 75% compression. Despite its high strength and stretchability, CGH2 is very durable. A CGH2 hydrogel can withstand 157 times the compressive strength of a CGH3 hydrogel, but not a CGH1 hydrogel, according to Figure 14a. 

Here are the mechanical performances of CGH1, CGH2, CGH3, and CGH4. The CGH2 hydrogel performed better when H_2_SO_4_ and graphene were added [49]. Moreover, the strength and modulus of the CGH1 and CGH2 hydrogels were superior to those of the CGH3 and CGH4 hydrogels after they were combined by in situ polymerization, followed by SO_4_^−2^ ions and H_3_O ions cross-linking [50,51]. 

There is a high degree of elasticity in the CGH2 hydrogel. According to Figure 14b, graphene levels increase with increasing tensile strength for the CGH2 and CGH4 hydrogels. Through surface graining and hydrogen interactions, graphene can effectively crosslink polymer chains in hydrogels. A high graphene content caused a strong hydrogen-bonding interaction, which resulted in cross-linking [51,52]. Under physiological conditions, amine bonds and hydrogen bonds form cross-links between an aldehyde or ketone group of polymers, which contribute to the strength of hydrogels [52]. As one of the most chemically and physically beneficial active carbon materials, hydrogels based on graphene exhibit excellent mechanical strength.

### 3.7. Proof of Concept

**Figure 15:**
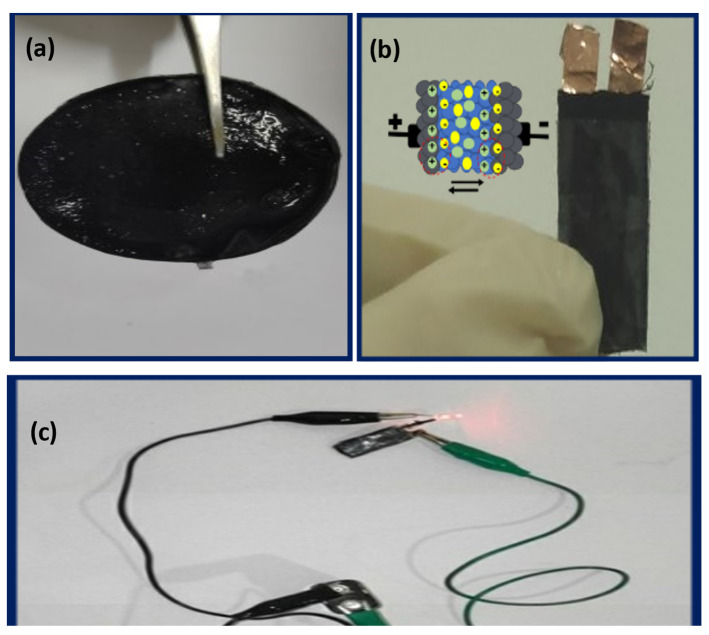
(**a**) Sample CHG2, (**b**) the setup for device fabrication by connecting two coated cotton/graphene electrodes with CHG2 hydrogel electrolyte in series, and (**c**) lighting of the LED lamp.

Sodium alginate, starch, and graphene-polymer cotton immersed in H_2_SO_4_ (CGH2) with an ionic conductivity of 13.9 × 10^−3^ S/cm and a thickness of 0.54 mm are shown in Figure 15. By configuring a fully flexible cotton-based supercapacitor with copper wire (Figure 13b), it can light a red LED at full power, as shown in Figure 15c, by connecting to a voltage source of 9 volts. The samples acted as supercapacitors by providing current to the LED and the LED was turned on with full intensity. In addition to exhibiting high energy storage density, the CGH2 supercapacitor exhibits excellent electrochemical performance. A hydrogel electrolyte developed with an open porous structure allows the electrolytes to interact effectively with the electrodes. A cotton/graphene electrode has been used successfully in supercapacitors to transport electrolyte ions or electrons quickly throughout the sandwich structure [53,54].

## 4. Conclusions

A novel cotton/graphene composite hydrogel electrolyte was developed using starch and sodium alginate to design a supercapacitor. X-ray diffraction analysis confirmed the sandwiching of the composite structure and revealed the crystalline peaks for the CGH samples, showing a high crystalline order for the sample CGH2. Morphology studies confirmed the porous 3D lattice structure, while elemental mapping described the appropriate dispersion of graphene in the hydrogel without any unwanted elements. Graphene functionalized with the hydrogels through imine-based linkers. The hydrogel containing pure cotton showed greater flexural strength, possibly due to the greater dispersion of the (smaller) pure cotton in the starch matrix state, and the good dispersion of cellulose nanocrystals led to a significant improvement in the module and the tensile strength of the hydrogel films. An electrochemical impedance analysis revealed that the ionic conductivity of the CGH2 hydrogel significantly increased up to 13.9 × 10^−3^ S/cm at 25°. The results suggest that the presence of graphene supports an increase in ionic conductivity and provides a smooth pathway for the transport of charge carriers and the polymer. Furthermore, CGH2 achieved the highest specific amplitude of 327 F/g at 3 mV/s during cyclic voltammetry (CV); a galvanostatic charge–discharge GCD analysis revealed 385.4 F/g at 100 mA/g current density. An electrochemical analysis demonstrated that a CGH cotton–graphene-based electrolyte hydrogel is electrically stable and presents reversible responses to electrochemical catalysts. The assembled device was used to light up a red LED lamp. The composite CGH2 hydrogel will have a significant impact on the design of the next generation of supercapacitors for the development of cells, due to its promising capacitive properties and excellent electrochemical stability. Therefore, composite hydrogel electrolytes will have wide applications in the field of energy storage.

## Figures and Tables

**Table 1:** Details of the samples concerning the cotton-to-graphene ratio.

System	
CGH1	Cotton (0.8 g) + graphene (0.2 g) + 5 mL hydrogel solution + 4 mL H_2_SO_4_.
CGH2	Cotton (0.8 g) + graphene (0.4 g) + 5 mL hydrogel solution + 4 mL H_2_SO_4_.
CGH3	Cotton (0.8 g) + graphene (0.2 g) + 5 mL hydrogel solution + 4 mL KCl.
CGH4	Cotton (0.8g) + graphene (0.4 g) + 5 mL hydrogel solution + 4 mL KCl.

**Table 2:** The specific capacitance (F/g), power density (W/Kg), and energy density (Wh/Kg) of the samples CGH1, CGH2, CGH3, and CGH4.

Cell	Specific Capacitance (F/g)	Power Density (W/Kg)	Energy Density (Wh/Kg)
CGH1	312.88	478.50	48.79
CGH2	390.75	499.99	50.99
CGH3	250.30	100.00	35.18
CGH4	333.50	100.10	52.25

## Data Availability

Research data can be provided on request to the corresponding author.
